# A Fault Diagnosis Model of an Electric Submersible Pump Based on Mechanism Knowledge

**DOI:** 10.3390/s25082444

**Published:** 2025-04-12

**Authors:** Faming Gong, Siyuan Tong, Chengze Du, Zhenghao Wan, Shiyu Qiu

**Affiliations:** 1 Qingdao Institute of Software, College of Computer Science and Technology, China University of Petroleum (East China), Qingdao 266580, China; gfaming@upc.edu.cn (F.G.); b21070003@s.upc.edu.cn (C.D.); 1907030128@s.upc.edu.cn (Z.W.); by1907040207@s.upc.edu.cn (S.Q.); 2 Shandong Key Laboratory of Intelligent Oil & Gas Industrial Software, Qingdao 266580, China

**Keywords:** electric submersible pump, fault diagnosis, knowledge engineering, deep learning

## Abstract

Electric submersible pumps (ESPs) are crucial equipment in offshore oilfield production. Due to their complex structure and the variable geological environments in which they work, ESPs are prone to a wide range of complex faults. Existing fault diagnosis models for ESP wells face several issues, including high subjective dependence, large sample data requirements, and poor adaptability to different geological environments. These issues lead to relatively low accuracy in ESP well fault diagnosis. To address these challenges, this paper integrates the mechanistic knowledge of ESP wells with their working parameters to construct a fault symptom inference model for ESP wells. A fault diagnosis model for ESP wells is formed by combining deep learning with an expert rule-based fault diagnosis method. The two models are connected in series to construct a mechanism knowledge-integrated ESP fault diagnosis model (MK-ESPFDM), achieving real-time and accurate diagnosis of faults in ESP wells. A series of experiments demonstrate that the proposed algorithm strategy can effectively improve the diagnostic accuracy of the model. It also reduces human subjectivity and enhances the model’s adaptability to different faults and geological environments. The research presented in this paper has reached a high level in the field of ESP well fault diagnosis.

## 1. Introduction

As a dominant technology in offshore oilfield production [1,2,3], ESP wells are crucial for oil recovery in wells characterized by complex geological conditions and high water content. However, the complex environment and well working conditions [4,5,6,7,8,9] lead to the complexity of the working status and the diversity of faults in ESP wells, making fault diagnosis of ESP wells a difficult problem. The purpose of this work is to improve the diagnostic accuracy of ESP wells, enable an algorithm model to diagnose various problems caused by environmental and geological changes, enhance the environmental adaptability of diagnostic algorithms, and thereby improve the production efficiency of oil fields [9,10,11].

Existing fault diagnosis methods for ESP wells face challenges such as strong domain specificity, limited fault data, and incomplete fault data records. These issues prevent data-driven deep learning models from fully learning and result in low diagnostic accuracy. Meanwhile, mechanism-based diagnostic methods are complex and lack timeliness. In response to these problems, this paper proposes a fault diagnosis method for ESP wells that integrates mechanistic knowledge. By characterizing and applying the mechanistic knowledge of ESP wells and combining it with the working status parameters of ESP wells, this method enables real-time and efficient fault diagnosis for ESP wells.

The existing working status diagnosis algorithm KG-BDA for ESP wells in oil production plants employs time-series analysis and Bayesian networks for fault diagnosis. This model constructs typical templates of fault feature parameter variation curves for various faults. It also applies a uniform pattern of time-series feature analysis to ESP wells across different geological conditions, leading to poor environmental adaptability. Moreover, the model uses Bayesian networks, with prior probabilities established through manual scoring by oilfield experts. This introduces significant human subjectivity into the diagnostic results. The model has many limitations and is not suitable for long-term application.

In summary, this paper aims to integrate the strengths of mechanistic and deep learning models, combine them with the knowledge graph [12,13,14,15,16] approach, and develop an efficient and accurate fault diagnosis algorithm for ESP wells. At the same time, it aims to demonstrate that the method of integrating mechanistic knowledge can improve the diagnostic accuracy of ESP wells and the adaptability to different geological environments.

The main work of this paper is as follows:

(1) Employ knowledge representation techniques to characterize and apply knowledge in the field of fault diagnosis for ESP wells, and integrate technologies such as reasoning machines and graph algorithms [17,18,19,20] with expert rules [21,22], semantic networks, and knowledge graphs to achieve diagnostic assistance.
(2) A multi-level fault diagnosis model based on the mechanistic knowledge and fault data of ESP wells has been developed. The model fully learns from the prioritized fault data samples and the variation characteristics of ESP well working status parameters, achieving efficient and accurate fault diagnosis for ESP wells.
(3) Experiments have been conducted to analyze the strengths and weaknesses of the proposed method in this paper, proving that it can improve the accuracy of the diagnostic algorithm for ESP wells and enhance its adaptability to various types of faults.

## 2. Materials and Methods

### 2.1. Related Work on ESP Fault Diagnosis

With the in-depth development of oil reservoir geology in oil fields, the working conditions and geological environments faced by oil wells are becoming increasingly complex, and the demands for oil production processes are also continuously increasing. Against this backdrop, the fault diagnosis of ESP wells used in offshore oil fields and high-water-cut wells has become a widely discussed issue among experts and scholars. The research on fault diagnosis of ESP wells is mainly divided into two categories: fault diagnosis methods based on mechanistic models and those based on data-driven approaches.

Mechanistic models are established based on a deep understanding of the mechanisms and principles underlying the ESP well system and its fault phenomena, through methods such as mathematical modeling and knowledge application. These models can reflect the essential laws and causal relationships of the ESP well system and fault phenomena. Therefore, the construction of mechanistic models requires substantial knowledge and expert experience to support them.

The current card method judges the working status of downhole equipment and potential faults by recording and analyzing the current changes during the working of the ESP well. The pressure build-up method involves closing the backpressure valve, applying a certain pressure at the wellhead, and observing the changes in the dynamic liquid level to analyze the causes of faults based on the pressure build-up curve. The system efficiency chart method collects parameters such as current, voltage, power, and flow during the working of the ESP well and judges the working status of downhole equipment and the faults that have occurred by drawing and analyzing the system efficiency chart. The expert system-based fault diagnosis method establishes and stores a large amount of domain-specific knowledge and rules, constructs a reasoning mechanism, and uses this knowledge to diagnose faults in ESP wells. These methods require a high level of professional knowledge for fault diagnosis, and their diagnostic results are significantly influenced by human subjectivity, often lacking real-time capability.

Data-driven fault diagnosis methods are techniques that leverage real-time and historical data collected during system working, combined with machine learning and deep learning technologies, to achieve fault detection and diagnosis. These methods rely on a large number of historical data samples and avoid the need for detailed knowledge of the physical structure of the equipment, making them suitable for fault diagnosis in complex industrial systems.

The vibration signal-based fault diagnosis method involves using vibration sensors to collect vibration signals from the working equipment of ESP wells. It achieves fault diagnosis for ESP wells by analyzing the vibration signals in both the time and frequency domains, combined with machine learning and deep learning techniques. The deep learning-based fault diagnosis method involves feature analysis of a large amount of historical fault data and uses deep learning models to automatically identify and predict equipment faults. These methods have strict requirements for the quantity and quality of training data, and they are often characterized by their black-box nature and lack of interpretability.

Specifically, Yang et al. [23] established extended Statistical Process Control rules based on the parameter variation patterns of ESP wells to achieve fault diagnosis. Yuan et al. employed deep learning methods to diagnose faults based on the prediction of ESP wellhead discharge. Du et al. used machine learning methods to construct a One-Class Support Vector Machine for the condition diagnosis of ESP wells. Qi et al. [24] proposed a fault diagnosis method for ESPs by integrating physical constraints with data-driven approaches using Long Short-Term Memory (LSTM). Sui et al. [25] introduced an attention mechanism into a Recurrent Neural Network (RNN) model to predict the energy consumption of ESP systems.

Based on these studies, despite the lack of fault data, this paper makes it possible to achieve integrated diagnosis of mechanistic knowledge and deep learning in the ESP well domain.

### 2.2. Mechanism Knowledge-Integrated ESP Fault Diagnosis Model

#### 2.2.1. Algorithm Framework

When diagnosing ESP faults, experts need enough evidence to conclude. These faults show up in the pump’s working parameters, called fault symptoms. To mimic expert diagnosis and ensure efficiency and interpretability, this article analyzes ESP working status parameters, infers fault symptoms, and uses them for diagnosis. It also builds a mechanism knowledge-integrated ESP fault diagnosis model using a knowledge graph of ESP well working status for comprehensive fault diagnosis.

**Figure 1:**
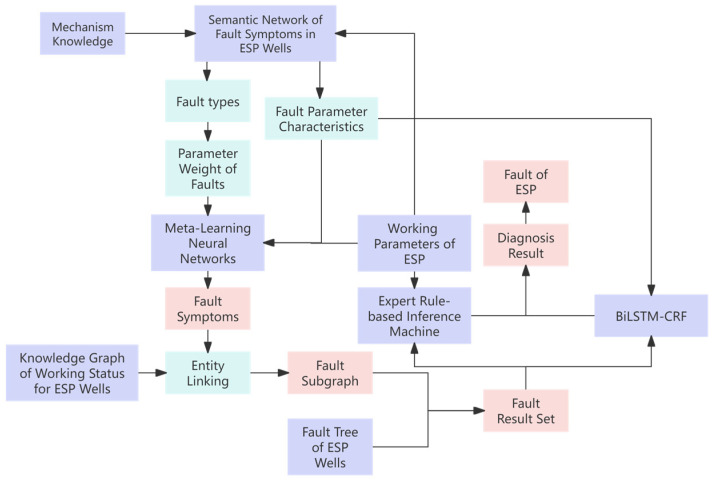
Overall architecture diagram of a fused mechanism knowledge–ESP fault diagnosis model.

Figure 1 shows the architecture of the MK-ESPFDM model. This algorithm includes a fault symptom inference model and a fault diagnosis model for ESP. In the fault symptom inference model, semantic networks and fault trees are used to represent and apply knowledge of ESP wells. This method is combined with ESP well parameters to infer fault symptoms. In the fault diagnosis model, a set of potential faults is obtained based on the inferred symptoms. Fault diagnosis is then carried out using an expert rule-based inference mechanism and a BiLSTM-CRF model. This approach implements a diagnostic inference pattern of “first gathering evidence and then diagnosing faults”.

#### 2.2.2. Representation of Mechanism Knowledge and Fault Symptom Inference for ESP Wells

**Figure 2:**
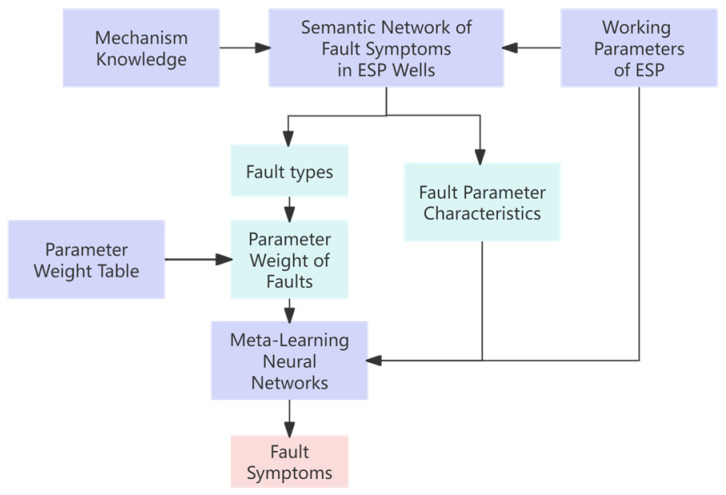
Framework diagram of fault symptom inference model for ESPs integrating mechanism knowledge.

This section mainly introduces the representation of the fault mechanism knowledge of ESP wells and the construction of the fault symptom inference model for ESP wells. Figure 2 shows the framework of the ESP fault symptom inference model.

(1) Representation of Mechanism Knowledge

Based on the automated data acquisition equipment of an oil production plant, real-time working parameters of the ESP wells are obtained, including three-phase current, voltage, wellhead oil pressure, wellhead temperature, wellhead back pressure, and wellhead casing pressure, totaling six parameters. In conjunction with the oil production plant’s yield calculation methods [26,27,28], the liquid production, oil production, and gas production, as well as the oil/gas ratio and liquid/gas ratio of the ESP wells, are determined.

These parameters reflect the working status characteristics of the ESP well in different aspects during working. For example, an increase in the value of the three-phase current causing the motor to overload shows two obvious fault symptoms: “increase in three-phase current” and “overload”.

This paper primarily categorizes fault types into four main categories: electrical system faults, mechanical system faults, environmental factors faults, and fluid-related faults and other faults.

**Figure 3:**
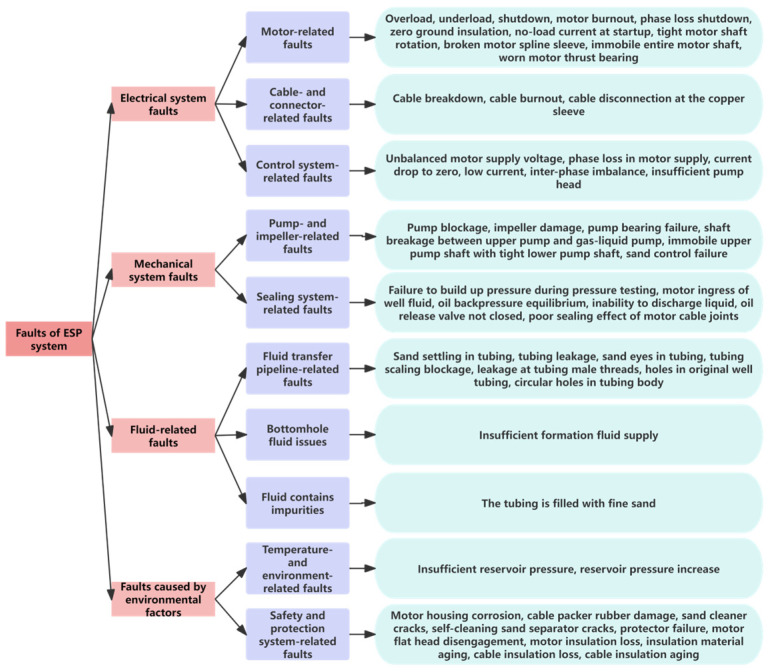
Fault tree of ESP.

**Figure 4:**
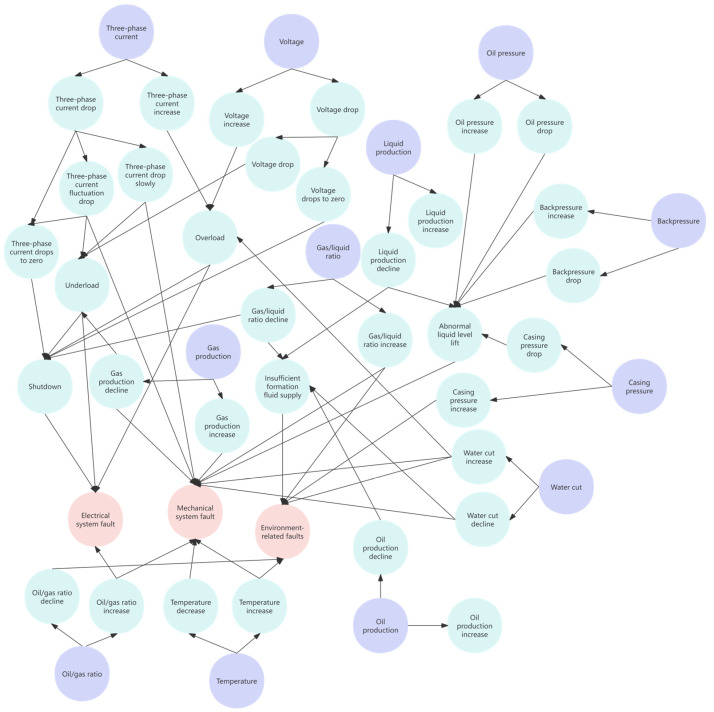
Semantic network for fault symptoms of ESP.

This study has extracted knowledge related to the mechanism structure and fault diagnosis of ESPs based on the publicly available literature on fault diagnosis of ESPs and the existing historical fault records of ESPs in oil production plants over the past decade. The fault mechanism knowledge of ESPs is classified according to fault types and fault symptom characteristics, forming 50 types of ESP well fault classifications suitable for actual production diagnosis. This study constructed a fault tree [29] for ESP wells and a semantic network [30]. Figure 3 shows the fault tree for ESPs, and Figure 4 shows the semantic network of fault symptoms for ESPs.

Different types of working parameters and fault indications have different associations with different fault types. This study employs probabilistic and statistical methods to organize and analyze the correlation between historical fault data and working parameters, forming a parameter weight table between ESP fault types and parameter types, as shown in Table 1.

For example, when an ESP experiences electrical system faults such as cable burning, the weight of electrical parameters like current and voltage is considered more significant than the pressure parameters related to the wellhead, with a weight of 0.48. The sum of the weights in each row of the table is 1.

Based on the real-time data of the ESP wells, this study conducts time-series analysis and classification of the changes in working status parameters over the past 60 time steps. The changes in parameters are categorized into four types: increase, decrease, fluctuation, and zero drop, which form a manifestation of the fault. Fault types are inferred using a semantic network based on these fault manifestations.

For example, if the voltage parameter shows an increasing trend over a certain period in the past, it is inferred as the fault manifestation of “voltage increase”. Based on the semantic network, the working status of the ESP well at this time is inferred to have experienced a fault of the type “electrical system fault”.

Subsequently, referring to the parameter weight table, this study retrieves the weights of the working parameters associated with this type of fault. These parameter weights are then calculated with the corresponding parameter sequences. The results of the calculations are concatenated with the fault parameter features to serve as the input for the subsequent model, thereby expanding the feature information contained in the model samples.

(2) A Fault Symptom Inference Model for ESP Wells Based on Meta-Learning Convolutional Shrinkage Neural Network

To address the challenges of limited historical fault data, poor document quality, and the complex working status of ESP wells, this paper constructed a meta-learning convolutional shrinkage neural network model.

**Figure 5:**
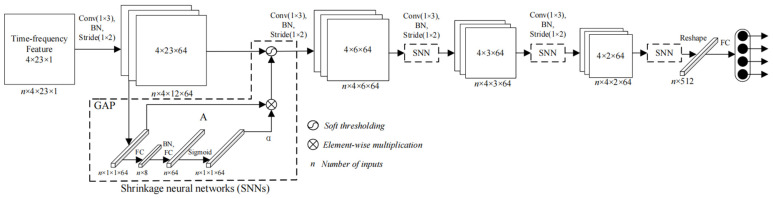
Structure diagram of meta-learning convolutional shrinkage neural network.

The model utilizes the idea of meta-learning [31,32,33] to analyze the time-series parameters of the working status of ESP wells and embeds a shrinkage neural network (SNN) [34,35] for thorough learning. Figure 5 is the architecture diagram of the inference model, which includes 1 input layer, 4 convolutional layers, 4 SNNs, and 1 fully connected layer.

In total, 80% of the historical fault dataset of the ESP wells is used as the training set $D^{tr}$ and 20% as the testing set $D^{ts}$. The support set and query set are separated in both the training set and the testing set using a similarity matching method.

The similarity calculation formula is (1).

(1) $$similarity(d_{i},d_{j})=\frac{d_{i}\cdot d_{j}}{\left\|d_{i}\right\|\cdot \left\|d_{j}\right\|}$$

In the formula, $d_{i}$ and $d_{j}$ are the working parameter sequences vectors of the ESP, and $\left\|d_{i}\right\|$ and $\left\|d_{j}\right\|$ are the norms of the vectors $d_{i}$ and $d_{j}$. Sample pairs with similarity scores above a predefined threshold are considered as similar samples and used as the support set. The support set is employed for the inner-loop training of the model, where model parameters are updated to learn the classification tasks of fault symptoms, thereby enabling fault symptom inference for the ESP on the query set. The remaining samples form the query set, which is used to evaluate the model’s adaptability to different fault symptom classification tasks of ESPs and further enhance the model’s few-shot learning capability.

For the support set $D^{trs}$ in the training set, the initial parameters θ are trained using the meta-learning convolutional SNN model. The optimal parameters $\theta^{*}$ are updated by summing all the losses in the query set $D^{trq}$. In the test set, the optimal parameters $\theta^{*}$ are fine-tuned using the support set, and the parameters $\theta_{i}^{*}$ are tested in the query set using the model, forming a process of “training–updating–testing”. Through meta-learning, the model can find a common parameter $\omega^{*}$ suitable for different fault symptom diagnosis tasks of ESP wells, and the calculation formula for the common parameter $\omega^{*}$ is Formula (2).

(2) $$\omega^{*}={argmin}_{\omega }\sum_{T_{i}\sim p\left(T\right),D_{I}\sim T_{i}}L_{i}\left(D_{i},\omega \right)$$

In the formula, $\omega^{*}$ represents the optimal parameter value for meta-learning training. ω represents the parameters of the meta model. $T_{i}$ represents all possible fault symptom inference tasks for ESPs. $p\left(T\right)$ represents the distribution of tasks. $D_{I}$ represents the support set obtained from sampling in task $T_{i}$, and $L_{i}\left(D_{i},\omega \right)$ represents the loss value obtained from training with support set $D_{i}$ on task $T_{i}$. Formula (2) aims to find the $\omega^{*}$, which is the parameter that minimizes the sum of losses obtained from training on the support set of all tasks.

By using meta-learning convolutional shrinkage neural networks, the model can construct a link between the working parameters of the ESP and the fault symptoms, combining with knowledge of the mechanism of ESP wells. This approach enables effective and accurate inference of ESP fault symptoms, especially useful when training data are relatively scarce.

The model also serves as an important demonstration of the integration of mechanistic knowledge of electric submersible pump wells with deep learning models in this paper.

#### 2.2.3. Knowledge-Integrated ESP Well Fault Diagnosis Model

**Figure 6:**
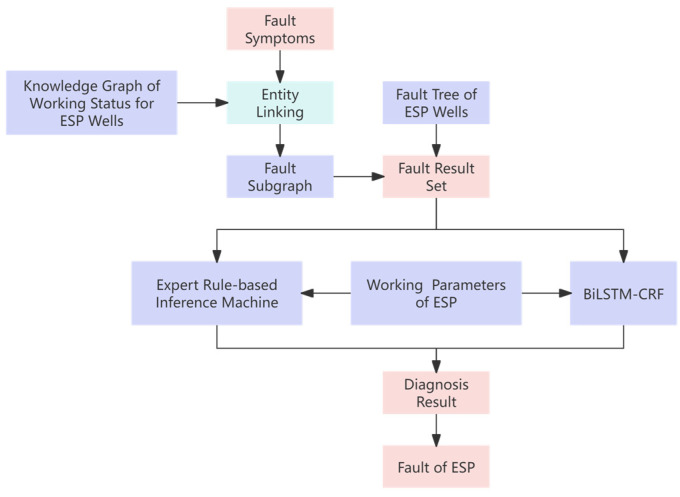
Framework diagram of knowledge-integrated ESP well fault diagnosis model.

This section mainly uses the inference results of fault symptoms of ESP wells, combines the knowledge graph of the ESP well working status, and screens out subgraphs and fault result sets. Through a dual diagnostic mode of neural networks and expert rules, the fault diagnosis of ESP wells is realized. Figure 6 shows the framework diagram of the knowledge-integrated ESP well fault diagnosis model.

(1) Fault Subgraph Query Based on Fault Symptom

The inferred fault symptoms and the fault symptom nodes in the knowledge graph are represented as string sets. The Jaccard similarity algorithm is used to calculate the similarity between strings, and the node with the highest similarity is selected for entity linking.

The formula for calculating Jaccard similarity is (3).

(3) $$J\left(A,B\right)=\frac{\left|A\cap B\right|}{\left|A\cup B\right|}$$

Among them, *A* and *B* represent the sets formed by strings that require similarity calculation. $\left|A\cap B\right|$ represents the size of the intersection between *A* and *B*, and $\left|A\cup B\right|$ represents the size of the union between sets *A* and *B*. The closer the Jaccard similarity is to 1, the more similar the two strings are.

After completing entity linking, the breadth-first search strategy is employed to obtain the neighboring nodes of each fault symptom node, which are then combined to form a subgraph. A spectral-based graph neural network (GNN) is utilized, leveraging the feature vectors of the subgraph nodes to compute the centrality of the node feature vectors. This process identifies the fault nodes with a higher probability of occurrence in the ESP when these fault symptoms coexist.

The GNN calculates the embedded representation of nodes through message passing. By continuously iterating and updating the representation of nodes, the embedding of nodes can capture the information of nodes and their neighboring nodes to reflect their importance in the network. Assuming the embedding representation of node *i* is $h_{i}^{\left(l\right)}$, where *l* represents the *l*-th layer where node *i* is located, the embedding update of node *i* can be calculated using the following Formula (4).

(4) $$h_{i}^{\left(l+1\right)}=f\left(h_{i}^{\left(l\right)},\left\{h_{j}^{\left(l\right)}|j\in N\left(i\right)\right\}\right)$$

In the formula, *f* represents the update function, $N\left(i\right)$ represents the set of adjacent nodes of node *i*, and $\left\{h_{j}^{\left(l\right)}|j\in N\left(i\right)\right\}$ represents the set of embedded representations of adjacent nodes of node *i*. When calculating the eigenvector centrality of a node, the eigenvalue is obtained by decomposing the Laplace matrix of the subgraph. The corresponding eigenvector is then derived, with the absolute value of its first non-zero element representing the node’s eigenvector centrality.

The formula is (5).

(5) $$C_{i}=\left|v_{1}\left(i\right)\right|$$

The 6 fault nodes with the highest feature vector centrality are ranked and filtered. In combination with the classification of fault types in the ESP fault tree, irrelevant faults are eliminated and the 4 most likely fault types are selected as the fault result set.

(2) Fault Diagnosis Based on BiLSTM and Expert Rules

By utilizing Bidirectional Long Short-Term Memory (BiLSTM) [36,37], the model can efficiently and accurately process a large amount of time-series data and diagnose faults in ESP wells based on known fault result sets and working parameters, ensuring good timeliness and accuracy of the model. Simultaneously, by incorporating expert rules of ESP fault diagnosis, an Expert Rule-based Inference Machine (ERIM) is constructed. This ERIM compensates for the neural network models’ low accuracy in identifying working parameters with different working statuses but similar time-series features, thereby enhancing the accuracy and reliability of working status recognition.

The input sequence for fault diagnosis consists of known fault symptom sets, fault result sets, and real-time working parameters of the ESP. BiLSTM is employed to infer and analyze the time-series features within the sequence. By extracting features from time series in both forward and reverse directions, BiLSTM effectively leverages the changing characteristics of working parameters for fault diagnosis. Additionally, the Conditional Random Field (CRF) module is incorporated as a multi-classifier to achieve fault conclusion inference.

Certain specific changes in the working parameters of ESPs are often inevitably linked to specific faults and may also have mutually exclusive relationships with some other faults. By constructing the ERIM, the strong correlation and mutual exclusion relationship between the fault symptoms and faults of ESPs are clarified. Leveraging historical fault data and expert knowledge from oil extraction plants, a set of correlation degrees between fault symptoms and faults is established to achieve the inference and diagnosis of ESP faults.

For example, in an ESP well, when the working status of “decreased production”, “temperature drop”, and “decreased three-phase current” occur simultaneously, the possible fault set screened by eigenvector centrality calculation includes four faults: “insufficient formation supply”, “pump outlet blockage”, “choked nozzle”, and “motor insulation failure”. In the fault diagnosis knowledge of ESP wells, a “pump outlet blockage” would cause an increase in three-phase current, which is mutually exclusive with “decreased three-phase current”. Therefore, using the ERIM, the fault of “pump outlet blockage” can be excluded. Similarly, the fault of “motor insulation failure” would lead to an increase in current values, which is also mutually exclusive with “decreased three-phase current” and is thus excluded by the inference model. After excluding the above two faults, based on the association degree set in the expert rules, it is further determined that the possibility of “insufficient formation supply” is higher than that of “choked nozzle”. Therefore, the final diagnosis result is “insufficient formation supply”.

Table 2 and Table 3 are partial representations of the association degree set and the mutual exclusivity table, respectively. The association degree relationship indicates the likelihood of a particular fault occurring when a certain fault symptom is observed. The mutual exclusivity relationship indicates that when a certain fault symptom is observed, a particular fault will definitely not occur.

The BiLSTM model and the ERIM diagnose the fault symptoms of the ESP well separately and output their respective diagnostic results and confidence levels. The fault result reasoning module will comprehensively evaluate the diagnostic results based on the confidence levels of the two models and select the conclusion with the higher confidence level as the final diagnostic result.

By driving the fault diagnosis through both the BiLSTM-based model and ERIM, the advantages of data-driven mathematical diagnosis can be fully utilized, combined with the mechanism diagnosis capabilities based on expert experience. This effectively improves the accuracy and reliability of ESP well fault diagnosis. Ultimately, this approach enables rapid identification of fault types, provides interpretable fault reasoning information, reduces the downtime of the ESP, and enhances the operational efficiency and safety of the ESP well system.

## 3. Experiments and Results Analysis

### 3.1. Experimental Setting

#### 3.1.1. Experimental Data

The corpus data for mechanism knowledge extraction come from 62 domestic and foreign studies, as well as 10 annual reports on ESP wells in oil plants, comprising a total of 7398 sentences. The knowledge graph in the field of ESP well working status is an existing knowledge base for oil production plants.

The fault diagnosis dataset used in this paper is constructed based on 1373 fault records from the offshore oil production platform of an oil extraction plant over the past 10 years. The basis for fault diagnosis is the working parameters of the ESP well. Therefore, this paper accesses the historical parameter database corresponding to the time nodes in the ESP well records. Based on the six parameters automatically collected by the sensors of the oil extraction plant before and after the fault, including three-phase current, voltage, wellhead oil pressure, wellhead temperature, wellhead back pressure, and wellhead casing pressure, the production calculations of the oil extraction plant are used to obtain the liquid production, oil production, gas production, oil/gas ratio, and liquid/gas ratio of the ESP well. These 14 working parameters form a time-series dataset, which serves as the data foundation for the model’s fault diagnosis.

**Figure 7:**
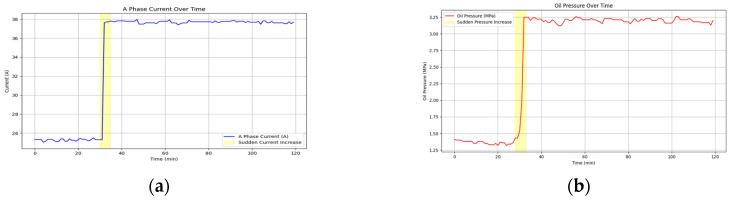
Visualization of the time-series working parameters of an ESP well: (**a**) the time-varying curve of three-phase current A; (**b**) the time-varying curve of oil pressure.

Figure 7 shows the time-series data visualization of three-phase current A and wellhead oil pressure during a particular fault in an ESP well.

Given that the working parameters of the ESP well remain stable during normal production, with values such as current, voltage, and liquid production all having a lower limit of 0, this paper opts for the Min–Max normalization method for data preprocessing. The normal working current, voltage, and wellhead oil pressure of each ESP well are used as the maximum values, while the 0 values of each parameter serve as the minimum values for Min–Max normalization processing. Considering that these parameters may encounter overload situations during the operation of the ESP well, this paper sets the upper limit of the normalized value to 1.5 based on data normalization. When the normalized value exceeds 1, the ESP well parameters are in an overload status.

The normalization method formula is as shown in Formula (6).

(6) $$x_{normalized}=\frac{x-x_{min}}{x_{max}-x_{min}}$$

The dataset was randomly shuffled and divided into three parts: 70% for training (961 fault records), 15% for validation (206 fault records), and 15% for testing (206 fault records).

#### 3.1.2. Meta-Learning Hyperparameter Information

This paper constructs a dataset based on the 27 common first-level fault symptoms of ESP wells, enabling the model to primarily infer these 27 first-level fault symptoms. The pre-training epochs are initially set to 10, the inner layer update steps to 5, the meta-learning rate to 0.001, the inner layer learning rate to 0.01, the batch size to 32, the number of classifications to 27, the K-shot learning number to 5, the query set size to 15, the number of input sequences to 14, and the time steps to 60; the number of fine-tuning epochs is 50. The model is compiled with the Adam optimizer and cross-entropy loss function, and early stopping is set to prevent overfitting; if the validation set loss does not improve for six consecutive epochs, training is stopped.

In this context, the K-shot learning number indicates that in meta-learning, for each new classification task, the model requires K samples to learn how to classify or predict these new tasks. The number of input sequences refers to the 14 types of working parameters of the ESP. The time steps mean that when inferring the fault symptoms of the ESP, the model takes into account the time-series data of the previous 60 time steps, i.e., the fault symptoms that occurred in the last 50 to 60 min before the inference.

The hyperparameters related to the meta-learning model are as shown in Table 4.

#### 3.1.3. Evaluation Criteria

This article focuses on 50 common types of faults in the actual production of ESPs, and conducts experiments using historical fault data of ESP wells in oil production plants. Given the model’s real-time analysis capabilities during practical application, this paper employs Recall@3 as an evaluation metric, which indicates the proportion of correct diagnostic results among the top three results identified by the model.

The calculation formula is (7).

(7) $$Recall@3=\frac{N_{3}}{N_{all}}$$

In the formula, $N_{3}$ is the number of predicted results that match the true results of the ESP fault in the first three predicted results, while $N_{all}$ is the total number of true diagnostic results.

To comprehensively evaluate the model’s performance, this paper uses the macro-average F1 score. The *F*1 *score* is the harmonic mean of precision and recall for each classification, and the macro-average F1 score is obtained by averaging the *F*1 *scores* across all classifications. Formulas (8)–(10) represent the calculations for *accuracy*, *precision*, and *F*1 *score*, respectively.

(8) $$Accuracy=\frac{TP}{TP+FP+TN+FN}$$

(9) $$Precision=\frac{TP}{TP+FP}$$

(10) $$F1score=\frac{2Accuracy\cdot Precision}{Accuracy+Precision}=\frac{2TP}{2TP+FP+FN}$$

In these formulas, True Positives (TP) indicates the number of samples that are actually positive and predicted as positive. False Positives (FP) indicates the number of samples that are actually negative but predicted as positive. False Negatives (FN) indicates the number of samples that are actually positive but predicted as negative. True Negatives (TN) indicates the number of samples that are actually negative and predicted as negative.

ESP well faults typically develop over 30 to 60 min from initial parameter changes to fault occurrence. The ability of an algorithm model to provide timely or early warnings based on ESP well parameters is a key performance metric. In the comparative experiment, the average diagnostic time from the first three ESP fault predictions is used to measure the model’s diagnostic speed.

### 3.2. Results and Analysis

#### 3.2.1. Comparison Experiment

This article employs a data-driven LSTM model to learn the sequence of working parameters before and after ESP faults, and compares it with the MK-ESPFDM to analyze the role of mechanism knowledge in ESP fault diagnosis. Additionally, diagnostic comparative experiments are conducted using the Knowledge Graph-based Bayesian Network Diagnostic Algorithm (KG-BDA) in oil extraction plants to determine if the MK-ESPFDM offers advantages in diagnostic accuracy. The experimental results are presented in Table 5.

In terms of the comprehensive diagnostic performance of the three models, MK-ESPFDM has surpassed LSTM and KG-BDA. However, the average diagnostic time of MK-ESPFDM is worse than that of LSTM.

**Figure 8:**
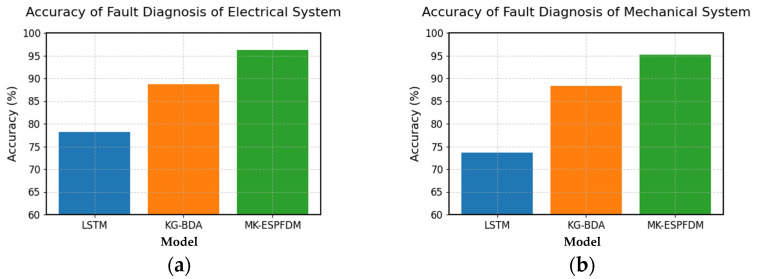

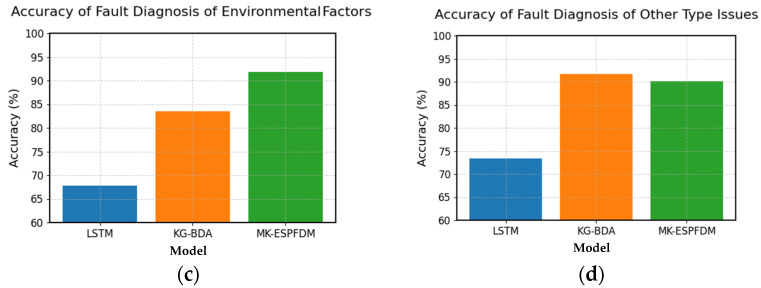
The Recall@3 comparison chart of the three diagnostic models for 4 major types of faults: (**a**) accuracy of fault diagnosis of electrical systems; (**b**) accuracy of fault diagnosis of mechanical systems; (**c**) accuracy of fault diagnosis of environmental factor issues; and (**d**) accuracy of fault diagnosis of other types of issues.

To better explore the fault diagnosis performance of the MK-ESPFDM model, this paper prepared 100 test samples for each of the four types of faults. Simulating the actual working status of the ESP well, the samples were input into the model step by step according to the time for fault diagnosis testing. The experimental results of the three models are shown in Figure 8.

From the experimental results, it can be seen that the accuracy of the three models in identifying electrical system faults and mechanical system faults is higher than that of the other two types of tasks. MK-ESPFDM shows better performance in most tasks, but its accuracy is slightly lower than that of KG-BDA when diagnosing other types of faults.

To more effectively explore how mechanism knowledge enhances model diagnostic accuracy and adaptability to diverse faults, this paper performs comparative experiments on the two sub-models of MK-ESPFDM: BiLSTM-CRF and ERIM. These experiments delve into the unique characteristics of each sub-model in diagnostic tasks and highlight the benefits of combining these models for fault diagnosis. The experimental results of fault diagnosis for the two sub-models of ESP are shown in Table 6.

Table 6 shows that BiLSTM-CRF performs better than ERIM in diagnosing ESP well faults. The integrated MK-ESPDFM model outperforms both individual sub-models.

To further investigate the differences in fault diagnosis among the sub-models, this paper conducted experiments on the four types of faults again. The results are shown in Figure 8.

**Figure 9:**
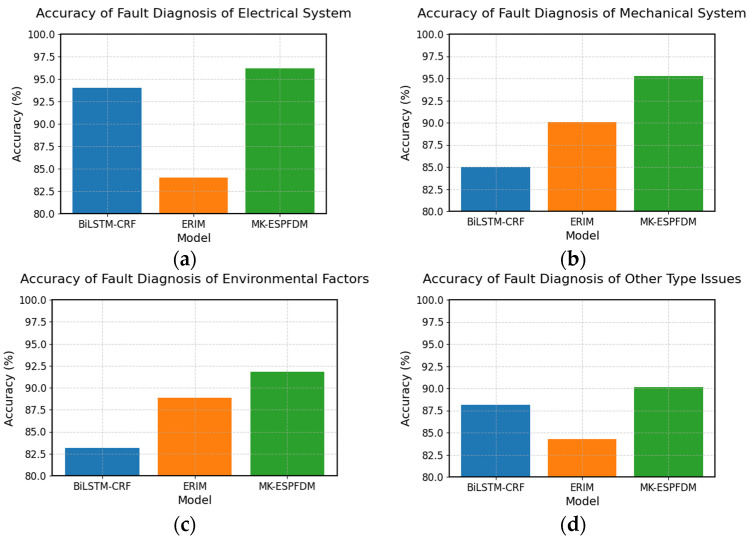
The Recall@3 comparison chart of sub-models in the diagnosis of four major categories of faults: (**a**) accuracy of fault diagnosis of electrical systems; (**b**) accuracy of fault diagnosis of mechanical systems; (**c**) accuracy of fault diagnosis of environmental factor issues; and (**d**) accuracy of fault diagnosis of other types of issues.

Based on the results in Figure 9, BiLSTM-CRF performs well in diagnosing electrical system faults and other types of faults, while ERIM has a clear advantage in diagnosing mechanical system faults and environmental factor faults. MK-ESPFDM outperforms the two sub-models in diagnosing all types of faults.

#### 3.2.2. Practical Application Effect

Given the model’s application in detecting abnormal working status in ESP production, it is essential to compare both diagnostic accuracy with KG-BDA and the model’s operational performance and adaptability to different well status.

ESP faults, particularly mechanical and environmental, often have long durations and early warning signs, unlike electrical faults, which occur rapidly without early warning, necessitating timely alarms to mitigate losses. This paper calculates warning times using mechanical and environmental fault examples and alarm times using electrical fault examples. MK-ESPFDM shows significant advantages in fault warning, with alarm times comparable to KG-BDA. Table 7 compares the warning and alarm times of the two diagnostic models applied in ESPs.

Table 5 shows that the proposed model significantly outperforms the existing oil extraction plant’s KG-BDA in warning of mechanical and environmental faults, while showing similar performance in warning of electrical faults.

## 4. Discussion

### 4.1. Discussion on the Results of Multiple Model Comparison Experiments

Based on the results in Table 5, MK-ESPFDM outperforms LSTM and KG-BDA in overall diagnostic performance. However, MK-ESPFDM’s average diagnostic time is longer than LSTM’s.

Analysis reveals that LSTM, due to its purely data-driven nature, does not require the use of a knowledge base for reasoning, thereby offering a significant advantage in terms of time performance. However, due to the scarcity of fault data, LSTM cannot be adequately trained, resulting in lower accuracy.

In contrast, the MK-ESPFDM model more fully leverages the mechanistic knowledge and working parameter characteristics of ESP wells, thus achieving better diagnostic accuracy than other models.

### 4.2. Discussion on the Diagnostic Results of Three Diagnostic Models for Four Types of Faults

Based on the experimental results in Figure 8, this paper concludes that electrical system faults and mechanical system faults occur frequently and provide ample data samples for algorithmic learning. Therefore, all three models demonstrate good accuracy in diagnosing these faults. However, the utilization of mechanistic knowledge varies among the models. Given the same data features, KG-BDA relies on a manually set scoring mechanism to achieve better performance than LSTM. In contrast, MK-ESPFDM, through multi-level fault diagnosis and full utilization of knowledge, achieves the best performance. This also proves that the strategy of integrating mechanistic knowledge in this paper effectively improves the algorithm’s accuracy and adaptability to different types of faults.

Conversely, for diagnosing other fault types with limited samples, KG-BDA’s reliance on prior probabilities derived from human expert evaluations results in better diagnostic accuracy than MK-ESPFDM. This suggests that while the research in this paper has diminished dependence on human subjective experience, it has consequently sacrificed some model performance in diagnosing other fault types.

### 4.3. Discussion on the Comparison of Experimental Results of Sub-Models

Based on the analysis of the experimental results in Table 6, this paper concludes that although BiLSTM-CRF outperforms ERIM in most cases, the integration of the two models significantly enhances the diagnostic accuracy of MK-ESPFDM. This outcome demonstrates that the strategy of incorporating mechanistic knowledge effectively improves the algorithm’s accuracy and also indicates that the proposed ERIM model in this paper has room for further improvement.

### 4.4. Discussion on the Diagnostic Results of Sub-Models for Four Types of Faults

The analysis of the experimental results in Figure 9 shows that BiLSTM-CRF can effectively extract temporal features when dealing with problems that have distinct parameter variation characteristics (such as changes in current and voltage), thereby achieving a high diagnostic accuracy. However, when diagnosing mechanical system faults and environmental factor faults, its performance may not be satisfactory due to the limited number of training samples.

The good performance of ERIM in diagnosing mechanical system faults and environmental factor faults indicates that the mechanistic model, through its rule-based and knowledge-driven reasoning, has a greater advantage in uncovering and inferring the potential relationships between fault symptoms and faults.

Therefore, integrating the strengths of both sub-models can enhance the adaptability of MK-ESPFDM to a variety of faults and environmental changes.

### 4.5. Comprehensive Analysis

MK-ESPFDM achieves efficient and accurate fault diagnosis of ESP wells by representing and applying the mechanism knowledge of ESP wells and combining deep learning methods.

Through a series of experiments in this paper, it can be confirmed that under the same training data status, MK-ESPFDM shows good diagnostic effects and alleviates the problem of scarce historical data.

This paper uses various knowledge representation techniques to avoid manually set prior probabilities and reduce the impact of human subjective factors.

Through comparative experiments of sub-models, this paper proves that the method of integrating mechanism knowledge and deep learning for diagnosis can effectively improve the accuracy and adaptability to various faults of the ESP well diagnosis algorithm.

This method and strategy can provide methodological support for the diagnosis of more other industrial equipment and management decision analysis, and has a good development prospect.

### 4.6. Limitations and Future Research Directions

When analyzing the limitations of MK-ESPFDM, this paper argues that the model has a clear dependence on the knowledge graph and knowledge base. This is a common issue faced by almost all algorithmic models that incorporate knowledge. However, unlike the existing models in oil plants, the knowledge-based algorithm relies on literature, knowledge, and equipment structure, rather than on prior probabilities set by humans. Therefore, the algorithm in this paper reduces the dependence on human subjectivity.

In future development, this paper proposes that the sources of fault data for ESP wells can be further enriched by incorporating multimodal data such as images, surveillance videos, and textual descriptions of faults. This multimodal approach can provide a richer set of data samples as support, thereby effectively alleviating the current issue of scarce historical fault data.

## 5. Conclusions

This paper investigates the fault diagnosis methods for ESP wells and thoroughly analyzes the existing problems in current fault diagnosis, such as the scarcity of historical fault data, significant influence of human subjectivity on diagnostic methods, and insufficient environmental adaptability. To address these issues, this paper proposes the MK-ESPFDM model, which integrates mechanistic knowledge with deep learning to achieve efficient and accurate fault diagnosis for ESP wells. A series of experiments demonstrate that incorporating mechanistic knowledge into the diagnostic algorithm enhances its accuracy and adaptability across various fault types. Therefore, the proposed algorithmic strategy in this paper can also be extended to the fault diagnosis of other industrial equipment, offering broad application prospects.

## Figures and Tables

**Table 1:** Parameter weight table.

Fault Type	Current- and Voltage-Related Fault Symptoms	Wellhead Parameter-Related Fault Symptoms	Production-Related Fault Symptoms	Yield Ratio-Related Fault Symptoms
electrical system faults	0.48	0.33	0.095	0.095
mechanical system faults	0.28	0.4	0.16	0.16
environmental factors faults	0.19	0.29	0.23	0.29
liquid-related faults and other faults	0.25	0.25	0.25	0.25

**Table 2:** Association degree set (partial).

Fault Symptom	Fault	Association Degree
Three-phase current increase	Overload	67.3%
Decreased production	Pump wear	43.1%
Three-phase current fluctuation	Control cabinet fault	34.4%
Three-phase current fluctuation	Motor insulation damage	36.8%
Gas/liquid ratio increase	Cavitation	52.9%
Voltage drop zero	Motor fault	85.2%

**Table 3:** Mutual exclusivity table (partial).

Fault Symptom 1	Mutually Exclusive Fault
Temperature drop	Cavitation
Three-phase current decreased	Motor insulation failure
Voltage drop	Motor insulation failure
Three-phase current decreased	Pump outlet blockage
Oil production increase	Pump leakage

**Table 4:** Meta-learning hyperparameter table.

Hyperparameters	Value	Description
EPOCHS_PRE	10	pre-training epochs
UPDATE_STEP	5	inner layer update steps
META_LR	0.001	meta-learning rate to 0.001
UPDATE_LR	0.01	inner layer learning rate to 0.01
BATCH_SIZE	32	batch size
NUM_CLASSES	27	number of classifications
K_SHOT	5	K-shot learning
QUERY_SIZE	15	query set size
NUM_FEATURES	14	number of input sequences
TIME_STEP	60	time steps
EPOCHS_FINETUNE	50	number of fine-tuning epochs

**Table 5:** Results of multiple model comparison experiments.

Model	Recall@3	F1 Score	Time
LSTM	73.26%	83.26%	33.2
KG-BDA	88.10%	87.74%	37.9
MK-ESPFDM	93.35%	89.93%	35.5

**Table 6:** Comparison of experimental results of sub-models.

Model	Recall@3	F1 score	Time
BiLSTM-CRF	87.56%	88.97%	34.1
ERIM	86.81%	87.32%	35.9
MK-ESPFDM	93.35%	89.93%	35.5

**Table 7:** Comparison of pre-warning and alarm performance of fault diagnosis models for ESP.

Model	Pre-Warning Time	Alarm Time
KG-BDA	42.9	32.8
MK-ESPFDM	38.3	32.6

## Data Availability

Restrictions apply to the availability of these data. Data were obtained from the Sinopec Shengli Oilfield Marine Oil Extraction Plant in Shandong Province, China, and are available from the authors with the permission of the Sinopec Shengli Oilfield Marine Oil Extraction Plant.

## Abbreviations

The following abbreviations are used in this manuscript:

BiLSTM	Bidirectional Long Short-Term Memory
BiLSTM-CRF	Bidirectional Long Short-Term Memory with Conditional Random Field
CRF	Conditional Random Field
ERIM	Expert Rule-Based Inference Machine
ESP	Electric submersible pump
FN	False Negatives
FP	False Positives
KG-BDA	Bayesian network-based electric submersible pump well fault diagnosis model
LSTM	Long Short-Term Memory
MK-ESPFDM	Mechanism Knowledge-Integrated Electric Submersible Pump Fault Diagnosis Model
RNN	Recurrent Neural Network
TN	True Negatives
TP	True Positives
